# Who should break the silos in One Health for global health security? an African perspective

**DOI:** 10.11604/pamj.2026.53.57.48576

**Published:** 2026-02-04

**Authors:** Frankline Sevidzem Wirsiy, Dine Roseline Dzekem, Nancy Berinyuy Tahmo, Ukenedo Collette Chika, Clinton Njakoi Kwemu, Eugene Vernyuy Yeika, Denis Ebot Ako-Arrey

**Affiliations:** 1 Africa Centres for Disease Control and Prevention, Addis Ababa, Ethiopia,; 2 Department of Epidemiology, College of Public Health, University of Nebraska Medical Center, Nebraska, USA,; 3 Cameroon Baptist Convention Health Board, Yaoundé, Cameroon,; 4 The Pandemic Fund Secretariat, 1818 H Street NW, Washington DC, USA,; 5 Shoreland Inc. at 933 N. Mayfair Rd., Milwaukee, WI 53226, Wisconsin, USA,; 6 Department of Social Sciences and Community Engagement, Rinda Ubuzima, Kigali, Rwanda,; 7 Department of Health Research Methods, Evidence and Impact, McMaster University, Hamilton, Ontario, Canada,; 8 Division of Epidemiology, Dalla Lana School of Public Health, University of Toronto, Toronto, Ontario, Canada,; 9 Health Systems and Programme Manager, Ministry of Public Health, Yaoundé, Cameroon,; 10 Catholic Relief Services, Brazzaville, Republic of the Congo

**Keywords:** One health, breaking silos, global health security, pandemic agreement, Africa

## Abstract

The One Health (OH) approach underscores the vital links between human, animal, and environmental health, advocating for collaborative strategies to strengthen global health security (GHS). However, disciplinary and institutional silos hinder the implementation of OH, particularly in Africa, where zoonotic diseases, antimicrobial resistance, climate change, and food insecurity have acute health impacts. This paper explores the question of “who should break the silos in One Health practice”, examining Africa's potential to drive GHS through a multisectoral governing OH strategy through a unified lens, with a focus on dismantling the silos. We discuss the roles of key stakeholders: governments, regional organizations, academia, and civil society, in leading cross-sectoral collaborations and building resilient, integrated health systems. Strengthening governance and fostering coordinated action across sectors are critical to realizing OH goals that not only protect Africa and the world at large but also contribute to global health resilience. An integrated governance model, with shared leadership and robust operational frameworks, is proposed as essential for advancing OH and securing health at all levels. Breaking silos in OH is not only vital for Africa's health security but is also central to global pandemic preparedness and response (PPR), particularly as the Pandemic Agreement now recognizes OH as a foundational pillar of its framework.

## Perspectives

### Introduction

The One Health (OH) approach aims to unify human, animal, and environmental health systems to prevent and control global health threats, including COVID-19, mpox, Ebola virus, and other zoonotic diseases, antimicrobial resistance (AMR), as well as climate change-related challenges affecting food security and reproductive health [1]. These systems and diseases are said to be managed if the Open Health Framework is fully incorporated while streamlining communication efforts towards knowledge and awareness of risk factors, route of transmission, effect, sound community health behaviors and prevention for resilient communities towards capping global health threats [2]. To advance the OH agenda, the quadripartite organization made up of the Food and Agriculture Organization of the United Nations (FAO), United Nations Environment Programme (UNEP), World Health Organization (WHO) and World Organization for Animal Health (WOAH) was created to drive the OH approach through stakeholders mapping, collaboration, financing, joint action plan, and OH scientific knowledge and evidence creation and exchange [3]. There is no doubt that the health security landscape has evolved, with increasing life expectancy from 58.1 years in 2000 to 63.5 years in 2019 due to a reduction in communicable diseases and improved surveillance systems [4].

Redefining WHO's definition of human health will facilitate actual implementation of OH approaches in the medical field, improve pandemic prevention, and reflect the many ways in which health determinants have evolved [5]. However, the implementation of the OH framework is often impeded by structural and operational silos across sectors, leading to fragmented responses that hinder timely and coordinated action [6]. This is further compounded by less investment, only 2%, directed towards health prevention for a continent with approximately one-quarter (¼) of the world's population [7]. As a result, breaking these silos is crucial for comprehensive global health security (GHS), as it enables coordinated surveillance, effective policy responses, and resource-sharing to combat diseases that cross human-animal-environmental boundaries. The interconnectedness of human, animal, and environmental health has long been recognised but not fully operationalised effectively, particularly in the African context, where complex socio-political challenges and resource constraints often obstruct interdisciplinary collaboration [8]. The OH concept, which advocates for this interdisciplinary collaboration and dependence, is essential for African countries, which face disproportionate health burdens from emerging and re-emerging diseases, food insecurity, and climate-related threats [8]. This paper addresses a crucial question: Who should take responsibility for dismantling these silos to ensure a cohesive and resilient OH approach in Africa? Identifying and empowering the stakeholders who can lead efforts to integrate human, animal, and environmental health systems is vital to advancing OH and securing the health of both African and global populations.

### The challenge of Silos in One Health

The term “silos” refers to isolated, non-collaborative structures across disciplines and institutions, which can impede a cohesive response to health threats. Silos within OH are largely due to differences in mandates, funding mechanisms, and regulatory environments among stakeholders such as ministries of health, finance, agriculture, and the environment. Additionally, conflicting priorities and a lack of cross-disciplinary knowledge hinder efficient cooperation for OH implementation [9]. OH is not only an African issue but a global concern, with Africa's success in adopting this approach directly impacting worldwide health security, particularly as the continent is prone to at least one infectious disease outbreak each year [10]. Let's take, for instance, the case of the recent mpox outbreak episodes in Africa and how they could potentially pose a global health threat, with cases being identified in other continents and countries, including China, Pakistan, the United Kingdom, and Canada [11]. The continuous episodes of such diseases, Ebola virus epidemic, the mpox epidemics, and the COVID-19 pandemic show how localised health crises can quickly become global concerns. By building OH in Africa, the continent can become a barrier to future pandemics and a paradigm for robust, cross-sectoral health systems.

### Consequences of Silos for global health security

Fragmentation in OH hinders the timely exchange of data, coordination of research, and limits sound tracking and comprehensive surveillance for zoonotic diseases and AMR [6]. This lack of synergy risks delayed responses, misallocation of resources, missed opportunities for preventive action, and delays in making health decisions even when laboratory findings are evident. Addressing these gaps requires an integrated framework that empowers stakeholders to collaborate effectively while respecting their unique expertise, especially as Africa's health systems remain plagued by structural issues that inhibit cohesive action.

### Key players who can break the Silos in Africa's One Health approach

***National governments and policymakers:*** African governments have seen their nations heavily affected by epidemics of different sizes and impacts, including the Cholera outbreaks with a case fatality ratio above the WHO-recommended 1% threshold [12] as well as the rising mpox cases in the region above the total number of figures recorded in 2025 [11]. These epidemics have increased African Heads of State's interest in OH issues and the health of their people. The growing interest is seen in their commitments towards championing the prevention of OH illnesses and financing efforts to curb them [13]. Examples of these investments and collaborations include the manufacturing of the first Polymerase Chain Reaction (PCR) test in Morocco [14] to help improve diagnoses and treatment as well as the transfer of vaccine and drug production technology in Africa Agreements will help improve equity in access to drugs and vaccines in the continent in time of need as well as create avenues for health financing, which are all pillar stone's of the region's GHS [15]. These key players, also being ministries of health, agriculture, finance, and environment, have not yet prioritised OH as compared to the harm it imposes on health security. Despite this, they and governments in general advance their efforts in developing formal mechanisms for collaboration to meet set goals. Additionally, legislative frameworks and funding allocations should be structured to advance common objectives and reinforce institutional accountability.

**African Union and regional bodies:** there are many agencies and actors in the space of OH, making valuable contributions. The establishment of the World OH Congress in South Africa and the African Food Safety Authority are significant initiatives [15]. The quadripartite (FAO/UNEP//WHO/WOAH) and international OH donors and partners, such as the Fleming Fund, Pandemic Fund, World Bank, and EcoHealth Alliance, are actively engaged at regional and country levels to support policy, strategy, and investment case for OH [3]. The OH operational tools, including the OH Zoonotic Disease Prioritisation (OHZDP) and the Surveillance and Information Sharing Operational Tool (SIS OT), are helping to develop a working-together culture for advocacy and policy change [16]. The African Union (AU), the Africa Centers for Disease Control and Prevention (Africa CDC), and Regional Economic Communities (RECs) also act as catalysts for OH by creating regional policies, guidelines, and monitoring frameworks. For example, in order to combat zoonotic illnesses, the AU, made up of 55 member states, supports the OH strategy, in which the human, animal, and environmental sectors collaborate to increase awareness, collect reliable data, carry out initiatives, and advance evidence-based policy and practice. Programs to combat zoonotic illnesses are being implemented by AU agencies such as the African Union Pan-African Veterinary Vaccine Center (AU-PANVAC), the Inter-African Bureau for Animal Resources (AU-IBAR), and the Africa CDC [8]. By aligning regional priorities and resources, these bodies drive and can help harmonise OH efforts across the continent. Furthermore, Africa CDC has spearheaded the creation and is assisting Member States in implementing a model called the Framework for OH Practice in National Public Health Institutes (NPHIs) [17], to help member states prevent and control zoonotic diseases throughout the continent.

**Academic and research institutions:** Africa's universities and research institutions, including the field epidemiology programs, play a leading role in breaking silos through transdisciplinary education and research. Training the next generation of scientists, policymakers, and practitioners in OH principles will foster a skilled workforce ready to tackle complex health issues. Collaborative research that crosses traditional disciplinary boundaries can also provide evidence to inform policy and practice. The OH scorecard [18] is an example of such collaborative research; developed to measure and promote integration among sectors such as public health, veterinary services, environmental health, and agriculture, the scorecard serves as a practical instrument in operationalising the OH approach at local, national, and global levels. Furthermore, the OH practice generated in West Africa by the academic group regarding surveillance and research has been making significant strides. Still, it has not been translated into action in the government settings. These indicate how important it is for the operationalisation of OH Framework to consider structural, regulatory, and administrative changes to create an enabling environment and sustainable financial mechanism through domestic funding.

***Community-based organisations and civil society:*** community organisations, often closest to vulnerable populations, are essential in implementing OH initiatives on the ground. These groups can play a critical role in promoting preventive health measures and responding to zoonotic disease outbreaks. Training and resource allocation, for example, through the pandemic fund [19] mechanism to these organisations will bridge gaps in service delivery and ensure that OH policies have tangible effects at the community level.

***Private sector and international partners:*** the private sector and international donors, including NGOs and global health organisations, have significant resources that can aid in breaking silos. By supporting public-private partnerships, donors can drive innovative approaches to health challenges and ensure the sustainability of OH initiatives. International partners should prioritise funding for integrative projects and infrastructure that foster collaboration across sectors.

### Strategies to break the silos: moving from theory to action

***Developing a One Health governance framework and ensuring political buy-in for the institutionalisation of the One Health practice in national public health institutes in Africa:*** a multisectoral governance model under a unifying lens is essential to coordinate roles and responsibilities among stakeholders in OH practice. The Pandemic Agreement enshrines OH as a critical tool for effective pandemic preparedness and response (PPR) [20]. Article 1b in the Pandemic accord includes a definition of OH for pandemic PPR, which aligns with the recognised definition of the OH High-Level Expert Panel (OHHLEP), an advisory body to the Quadripartite. OH's significance is further reinforced by Article 5, which establishes flexible commitments encouraging nations to address the drivers of pandemics through consideration of the human-animal-plant-environment interface within their respective health systems, legal and practical capacities [3]. Yes, it's worth applauding the quadripartite for OH collaborative initiatives between four international agencies that work to improve the health of humans, animals, plants, and the environment i.e.: Food and Agriculture Organization of the United Nations (FAO), World Organization for Animal Health (OIE), United Nations Environment Programme (UNEP), and World Health Organization (WHO). This enhances OH collaboration with relevant stakeholders, ensuring a more holistic, efficient, and sustainable public health system across the continent, strengthened by National Public Health Institutes. The biggest question is whether there is political buy-in for the implementation of these frameworks! The effectiveness of implementing frameworks on the OH approach/governance hinges on strong political commitment from the AU heads of state. Political buy-in ensures the allocation of resources, policy alignment, and sustained collaboration across sectors such as health, animal agriculture, and the environment. Without this commitment, the approach may lack the necessary support for impactful action, risking fragmented efforts and incapacitating the technical efforts of the OH approach implementation. Adequate political will can drive cohesive strategies, harmonised regulations, and regional cooperation, ultimately advancing the OH agenda across the continent.

***Cross-sectoral data integration and surveillance systems:*** establishing an interoperable data system for OH surveillance is essential. Data-sharing protocols must be developed to ensure that information flows smoothly across human, animal, and environmental health systems. International organizations can support this effort by providing technical guidance and standardising data-sharing protocols. Integrated surveillance systems would improve early detection and coordinated response to emerging threats.

***Capacity-building and workforce development:*** to overcome silos, stakeholders must invest in capacity-building for a workforce skilled in interdisciplinary approaches. Universities and training institutions should develop a OH curricula that emphasize cross-sectoral skills, while governments and international organisations provide continuous professional development programs. Workforce development initiatives should prioritise equipping health professionals with knowledge of zoonotic disease epidemiology, AMR, environmental health, and social sciences.

***Encouraging resource sharing, respectful and action-oriented public-private partnerships:*** resource sharing, respectful and action-oriented Public-Private Partnerships (PPPs) can accelerate OH initiatives by leveraging private sector resources and expertise. Governments should incentivise PPPs focused on OH issues like AMR and zoonotic diseases. These partnerships would benefit from clear legal frameworks that outline roles, responsibilities, and risk-sharing mechanisms.

***Fostering community engagement and civil society involvement:*** effective OH implementation requires active engagement with communities that interact closely with animals and the environment. Civil society and community groups should play a key role in educating the public on health risks and preventive measures. By fostering local knowledge-sharing and advocacy, these groups can enhance surveillance and support for OH policies.

### Conclusion

Breaking the silos in Africa's OH approach is not the responsibility of a single actor; it requires a concerted effort among governments, regional bodies, academia, community groups, civil society and international partners. In the same light, breaking the silos that hinder the OH approach in Africa is a critical step toward enhancing both continental and global health security. Through coordinated policy-making, resource sharing, and capacity building, Africa can champion a sustainable and effective OH model. The stakes are high; not just for the continent of Africa but for global health security. As Africa strengthens its OH response, it sets a precedent for resilience, preparedness, and collaboration on a global scale. Africa's leadership and experience in addressing interconnected health challenges provide an essential perspective for shaping the future of OH and establishing a more secure, resilient global health landscape. By implementing a multisectoral governance model under a unified lens, enhancing data-sharing, and fostering public-private partnerships, the global health community can enhance OH implementation for robust global health security. Stakeholders must prioritise breaking silos, as it is only through true interconnectivity that the full potential of OH can be realised to prevent and mitigate global health threats. By investing in bridging structural gaps, local capacity, and scaling cross-sectoral coordination, Africa can transform from a hotspot of emerging health threats like cholera, mpox, etc., to a hub of health resilience and innovation. Moving forward, breaking silos in OH is not only critical to strengthening Africa's health security but also central to global pandemic preparedness and response (PPR), particularly as the Pandemic Agreement now recognises OH as a foundational pillar of its framework. Moving from rhetoric to action is urgent. Only through dismantling silos can Africa, and the world, fully harness the potential of OH to prevent, detect, and respond to the next outbreak, epidemic, and pandemic threat.

## References

[1] World Health Organization (WHO) One Health.

[2] Segerberg A, Bennett WL (2011). Social media and the organization of collective action: Using Twitter to explore the ecologies of two climate change protests. The Communication Review.

[3] World Health Organization (WHO) Quadripartite call to action for One Health for a safer world.

[4] World Health Organization (WHO) World health statistics 2025: Monitoring health for the SDGs, Sustainable Development Goals.

[5] Lefrançois T, Angot JL, Autran B, Bukachi SA, Claverie de Saint-Martin E, Giraudoux P (2025). A new definition of human health is needed to better implement One Health. Lancet.

[6] Yopa DS, Massom DM, Kiki GM, Sophie RW, Fasine S, Thiam O (2023). Barriers and enablers to the implementation of One Health strategies in developing countries: a systematic review. Front Public Health.

[7] Flying Doctors and Stears Data Impact Investing and Healthcare Financing in Africa.

[8] Alimi Y, Wabacha J (2023). Strengthening coordination and collaboration of One Health approach for zoonotic diseases in Africa. One Health Outlook.

[9] Dos S, Ribeiro C, van de Burgwal LHM, Regeer BJ (2019). Overcoming challenges for designing and implementing the One Health approach: A systematic review of the literature. One Health.

[10] Odey M Seven Common Communicable Diseases in Africa: Causes and Control.

[11] World Health Organization (WHO) Mpox: Multi-country External Situation Report no.53.

[12] Bekele BK, Uwishema O, Bisetegn LD, Moubarak A, Charline M, Sibomana P (2025). Cholera in Africa: A Climate Change Crisis. J Epidemiol Glob Health.

[13] Africa CDC Partnerships for African Vaccine Manufacturing (PAVM) Framework for Action.

[14] Africa CDC Morocco's First Homegrown PCR Test for mpox Gets Africa CDC Nod.

[15] Africa CDC The change in African vaccine manufacturing landscape.

[16] CDC One Health One Health Zoonotic Disease Prioritization (OHZDP).

[17] Africa CDC Framework for One Health Practice in National Public Health Institutes.

[18] Reperant L, Mackenzie J, Venter M, Mulumba M, Osterhaus A (2025). Scientific highlights of the 8th world One Health Congress, Cape Town, South Africa 2024. One Health Outlook.

[19] The Pandemic Fund The Pandemic Fund (Overview).

[20] Food and Agriculture Organization of the United Nations (FAO) The WHO Pandemic Agreement - A landmark for One Health.

